# Concurrent pulmonary zygomycosis and *Mycobacterium tuberculosis *infection: a case report

**DOI:** 10.1186/1752-1947-1-17

**Published:** 2007-05-03

**Authors:** Tejal Patel, Ian J Clifton, Jack A Kastelik, Daniel G Peckham

**Affiliations:** 1 Department of Respiratory Medicine, St James's University Hospital, Leeds, UK

## Abstract

A non-smoking 77-year old gentleman of Indian origin was admitted with a 4-month history of intermittent night sweats, haemoptysis and 6 kg of weight loss. CT scan of thorax demonstrated a 2.5 cm mass in the right middle lobe with multiple small nodules within the right lung and confirmed the presence of mediastinal and hilar lymph nodes.

Fibreoptic bronchoscopy demonstrated a distorted right main bronchus, anterior shift of the right upper lobe and occlusion of the right middle lobe bronchus with a black necrotic ulcer. *Mycobacterium tuberculosis *was found in the bronchoalveolar lavage and histology demonstrated numerous fungal hyphae with a morphological appearance of zygomycetes within necrotic areas of tissue. Medical management with anti-fungal and anti-mycobacterial treatment was instigated as the patient's pre-existing IHD did not permit surgical intervention. Subsequently CT imaging following completion of therapy demonstrated improvement of the mass and a resolution of the associated nodules. The patient has been followed for 6 months to date and there has been no recurrence of symptoms. Recent bronchoalveolar lavage cultures have been negative for *M. tuberculosis *and zygomycetes.

## Case presentation

A non-smoking 77-year old gentleman of Indian origin was admitted with a 4-month history of intermittent night sweats, haemoptysis and 6 kg of weight loss. He had no past history of *Mycobacterium tuberculosis *infection but suffered from significant ischaemic heart disease (IHD). There was no history of diabetes mellitus and random blood glucose levels were normal. Testing for human immunodeficiency virus (HIV) was not undertaken as the patient was not felt to be at risk for HIV infection. Clinical examination was unremarkable apart from low grade pyrexia. He was retired and had recently arrived in the United Kingdom from India.

Routine laboratory investigations were normal apart from an elevated CRP at 22.1 mg/L. Chest x-ray revealed bilateral hilar lymphadenopathy and a mass in the right lower zone. CT scan of thorax demonstrated a 2.5 cm mass in the right middle lobe with multiple small nodules within the right lung and confirmed the presence of mediastinal and hilar lymph nodes.

**Figure 1:**
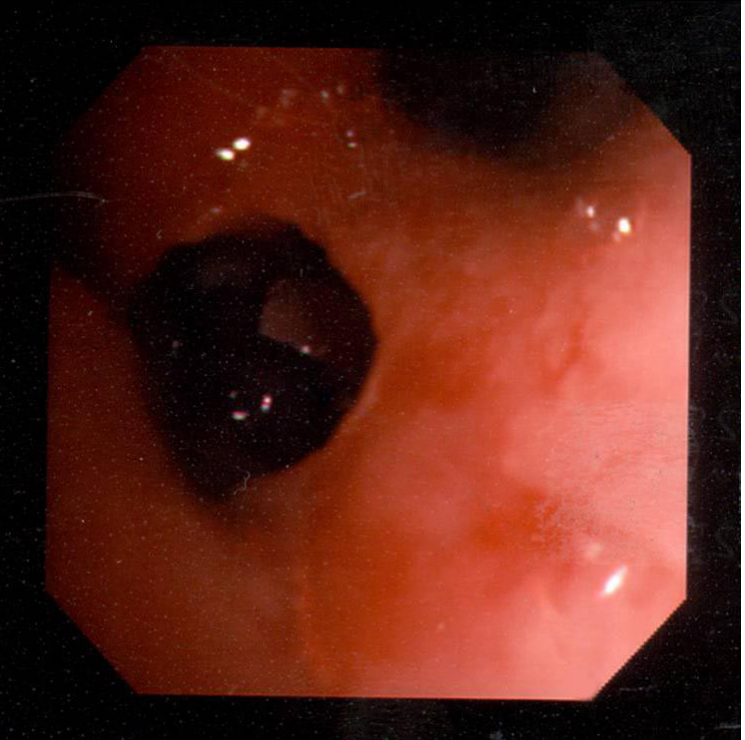
Black necrotic material in right middle lobe bronchus.

Fibreoptic bronchoscopy demonstrated a distorted right main bronchus, anterior shift of the right upper lobe and occlusion of the right middle lobe bronchus with black necrotic material (See Figure 1). Acid and alcohol fast bacilli (AAFB) were visible on microscopy in the bronchoalveolar lavage (BAL) fluid and were subsequently identified as *M. tuberculosis*. Histological examination of endobronchial biopsies taken from the necrotic material showed numerous fungal hyphae with a morphological appearance of zygomycetes within necrotic areas of tissue. Fungal cultures were negative; therefore anti-fungal sensitivity testing could not be performed.

Medical management was instigated as the patient's pre-existing IHD did not permit surgical intervention. Intravenous liposomal amphotericin (Ambisome, Gilead) at a dose of 3 mg/kg and standard four drug anti-mycobacterial regimen consisting of rifampicin, isoniazid, pyrazinamide and ethambutol was commenced. Following three weeks of therapy the intravenous liposomal amphotericin was changed to oral itraconazole (Sporanox, Janssen-Cilag) 200 mg once daily, which was increased to 200 mg twice daily following low therapeutic monitoring. Subsequent itraconazole levels were within the therapeutic range.

The patient completed 6 months of oral anti-fungal treatment. Due to concerns on a follow-up CT scan regarding lack of resolution of the multiple nodules 18 months of anti- mycobacterial chemotherapy was administered. Subsequently CT imaging following completion of anti-mycobacterial chemotherapy demonstrated improvement of the mass and a resolution of the associated nodules. The patient has been followed for 6 months to date and there has been no recurrence of symptoms. Recent BAL cultures have been negative for *M. tuberculosis *and zygomycetes.

## Conclusion

Zygomycetes are the third most common invasive fungal infection in humans after *Aspergillus sp*. and *Candida sp*. Inhalation of the spores from the environment is thought to be the primary mode of transmission of zygomycetes [1] with the lungs being the second commonest site of infection [2,3].

Pulmonary zygomycosis is 2 to 3 times more common in men than women [4,5]and the main risk factors include diabetes mellitus, haematological malignancy, renal insufficiency and solid organ transplantation. Pulmonary zygomycosis has been rarely reported in the absence of recognised risk factors [6,7]. Up to 32% of patients presenting with zygomycosis have been observed to have a concurrent infection which is usually bacterial in origin [4]. In some cases zygomycetes may infect lung cavities following pulmonary tuberculosis [8]. There is only one report of pulmonary zygomycosis and *M. tuberculosis *infection occurring simultaneously in a patient with acute myeloid leukaemia [9].

Pulmonary zygomycosis usually presents as a diffuse pneumonia causing cough, fever and haemoptysis. Involvement of the mediastinal structures can occur as does distant haematogenous spread. Chest X-ray typically shows consolidation or the presence of discrete masses. Chest CT scans can reveal additional abnormalities and cavitation in 26% and 40% of cases respectively [10]. Bronchoscopy may be useful in establishing the diagnosis of zygomycosis via BAL or transbronchial biopsy. The endobronchial findings of zygomycosis include the presence of granulation tissue, gelatinous tissue, stenosis and a necrotic ulcer [11]. Collins et al reviewed the published cases of endobronchial zygomycosis and found that the right bronchial tree was more commonly involved, and postulated the possibility of inhalation or aspiration of material may be important in the pathogenesis of the condition [11]. Histology is often required to establish the diagnosis which typically shows non-septated right angle branching-shaped hyphae [3]. Combined surgical and medical treatment of zygomycosis has a reported mortality of 45%, compared to medical treatment alone which has a mortality of 70–80% [5,10]. Treatment of zygomycosis consists of the prompt instigation of amphotericin treatment, preferentially combined with surgical resection of the necrotic tissue. Oral azoles have little activity against zygomycetes; however there are anecdotal reports of azoles having some benefit [12-14]. Posaconazole, a new triazole maybe of some benefit in the treatment of patients with zygomycosis [15]. The main determinant of mortality relates to the nature of the underlying disease.

To our knowledge this is the first report of concurrent pulmonary zygomycosis and *M. tuberculosis *infection occurring in a patient with an absence of recognised risk factors. Despite surgical intervention being precluded due to IHD, medical therapy has resulted in a cure.

## Abbreviations

AAFB Acid alcohol fast bacilli

BAL Bronchoalveolar lavage

CRP C-Reactive protein

HIV Human immunodeficiency virus

IHD Ischaemic heart disease

## Competing interests

The author(s) declare that they have no competing interests.

## Authors' contributions

All authors read and approved the final manuscript.
